# Multi‐Component and Cognitive Skills Training in Mild Cognitive Impairment: Outcomes from a South American Study

**DOI:** 10.1002/alz.091916

**Published:** 2025-01-09

**Authors:** Maria Eugenia Martin, Carlos Martinez, Iris Worff, Maria Agustina Belucci, Greta Keller, Micaela Maria Arruabarrena, Nicolas Corvalan, Maria Florencia Clarens, Ricardo Allegri, Lucia Crivelli

**Affiliations:** ^1^ Fleni, Buenos Aires, CABA Argentina

## Abstract

**Background:**

Dementia represents a significant health challenge, with evidence suggesting that it can be potentially delayed or prevented through non‐pharmacological interventions. There are different types of non‐pharmacological interventions for Mild Cognitive Impairment (MCI). The Aging Well through Interaction and Scientific Education (AgeWISE) Program is a notable cognitive intervention designed to educate both individuals experiencing normal aging and those with age‐related diseases. It offers strategies for managing age‐related cognitive changes and provides skill training.

**Goals:**

This study aims to evaluate the effects of a multi‐component psychoeducation intervention (AgeWise) in patients with MCI, focusing on the aging brain, lifestyle factors associated with successful brain aging, and strategies to compensate for age‐related cognitive decline.

**Method:**

A group of 25 amnesic and amnestic‐multidomain MCI patients (mean age: 75.26±5.5, 65.38% female, education level: 14.46±2.8, MOCA: 22.96±2.91) were recruited from a Memory Clinic in Buenos Aires. Patients underwent 10 treatment sessions based on the AgeWISE program. Pre‐treatment (week 0) and post‐treatment (week 10) assessments included the Multifactor Memory Questionnaire (MMQ) for various memory dimensions and the Depression, Anxiety, and Stress Scale (DASS‐21) to assess mood. The Wilcoxon‐Mann‐Whitney model was used to analyze the impact of treatment on each outcome.

**Result:**

The pre and post‐treatment measures were compared, revealing an improvement in the total DASS‐21 (P = 0.05), with a significant reduction in depression (DASS‐21‐Depression P = 0.02). While changes in the MMQ scale did not reach significance, there was a trend towards improved satisfaction with memory performance (P = 0.083).

**Conclusion:**

In regions with limited access to clinical and pharmacological trials, non‐pharmacological interventions emerge as a viable option for MCI patients. The AgeWISE intervention specifically demonstrated improvements in mood among MCI patients, with significant changes were observed in specific items (DASS‐21‐Total and Depression) related to initiative, tolerance for interruptions, and enthusiasm, suggesting positive outcomes for individuals undergoing this intervention. These findings highlight the potential benefits of non‐pharmacological interventions for individuals with MCI.

